# Human Gordon Holmes Syndrome modeling in mice reveals essential function of RNF216 ubiquitination in spermatogenesis and male fertility

**DOI:** 10.1016/j.gendis.2026.102056

**Published:** 2026-01-29

**Authors:** Jeffrey M. Mann, Chao Wei, Xiaoyuan Yan, Huirong Xie, Elena Y. Demireva, Chen Chen

**Affiliations:** a Department of Animal Science, Michigan State University, East Lansing, MI 48824, USA; b Reproductive and Developmental Sciences Program, Michigan State University, East Lansing, MI 48824, USA; c Transgenic and Genome Editing Facility, The Research Technology Support Facility, Institute for Quantitative Health Sciences & Engineering, Michigan State University, East Lansing, MI 48824, USA; d Department of Obstetrics, Gynecology and Reproductive Biology, Michigan State University, Grand Rapids, MI 49503, USA

Gordon Holmes syndrome (GHS) is a devastating disease characterized by neurological and reproductive dysfunction, including ataxia, dementia, and hypogonadism. Currently, patients have few positive outcomes, and little is known about disease etiology and progression at the cellular and molecular levels. Whole-genome sequencing of GHS patients identified mutations in Ring finger protein 216 (RNF216/TRIAD3), an E3 ubiquitin ligase, and OTU domain-containing protein 4 (OTUD4), a deubiquitinase.^1^ Patients harboring either homozygous or compound heterozygous mutations of *Rnf216* alone presented both neurological and reproductive phenotypes, suggesting *Rnf216* is the primary underlying genetic factor in GHS. Consequently, additional case reports have described GHS to be associated with *Rnf216* mutations near the catalytically active ubiquitination region, the Ring-between-Ring (RBR) domain. The RBR domain classifies RNF216 as a specialized subset of E3 ubiquitin ligases due to its mechanism that utilizes both RING domains to coordinate protein ubiquitination. This is also observed in the RBR family member, Parkin, linked to neurodegenerative Parkinson's disease. At the molecular level, RNF216 exhibits an *in vitro* preference for non-canonical protein ubiquitin chain linkages, lysine-11 (K11) and K63, which are associated with signaling and DNA-damage pathways rather than proteasomal degradation via canonical K48-linked ubiquitination, suggesting that these downstream pathways may be involved in the molecular etiology of GHS^2^, ^3^, ^4^. In addition, analysis of human GHS-associated RNF216 mutations near the catalytically active cysteine in the RBR domain showed that these mutations completely abolished E3 ubiquitin ligase activity *in vitro*, rendering RNF216 catalytically inactive.^2^^,^^4^ Our group was first to report *Rnf216* gene knockout (*Rnf216 KO*) in mice, which resulted in disrupted spermatogenesis and male infertility, highlighting the essential role of RNF216 in male reproduction.^5^ However, the key molecular mechanism underlying RNF216-related GHS and its associated reproductive dysfunction remains unclear. To gain a better understanding of the human GHS RNF216 mutation in male reproduction, we generated a novel transgenic mouse model harboring the E3 ubiquitin ligase-inactivating mutation observed in GHS patients.

Using CRISPR-Cas9 genome editing, we introduced the human GHS-associated mutation RNF216 R751C into exon 15 of the *Mus musculus Rnf216* gene, corresponding to RNF216 R739C in the mouse protein (mouse numbering used hereafter unless otherwise noted) (Fig. 1A and B; Fig. S1). This mutation lies six residues downstream of the catalytically active cysteine (C733 in mouse; C745 in human), and as confirmed by sequence alignment, this residue is conserved between humans and mice (Fig. 1B). Sanger sequencing and PCR confirmed the correct mutation in transgenic founder (F0), heterozygous offspring (F1), and homozygous mutant mice (F2) (Fig. S1C and S1D). To assess the impact of the RNF216 R739C GHS mutation on male fertility, wild-type and homozygous mutant male mice (*Rnf216 R739C/R739C*, hereafter referred to as “*Rnf216 GHS*”) were bred with wild-type females for four months. While wild-type males exhibited normal fertility (6.8 pups/litter ± 0.22; number of different male mice tested, *n* = 3), *Rnf216 GHS* males were infertile (0 pups/litter ± 0; number of different male mice tested, *n* = 3) (*p* = 0.0001) (Fig. 1C). The appearance of *Rnf216 GHS* mice was similar to wild-type littermates (Fig. 1D). However, adult *Rnf216 GHS* males exhibited significantly reduced testis size (0.029 g ± 0.001; number of mice, *n* = 10) compared with wild-type males (0.099 g ± 0.002; number of mice, *n* = 8) (*p* = 3.2e-11) (Fig. 1E and F). In contrast, female *Rnf216 GHS* mice displayed no apparent abnormalities in gross reproductive development and remained fertile (*p* = 0.41) (Fig. S2), resembling *Rnf216 KO* females.^5^

**Figure 1:**
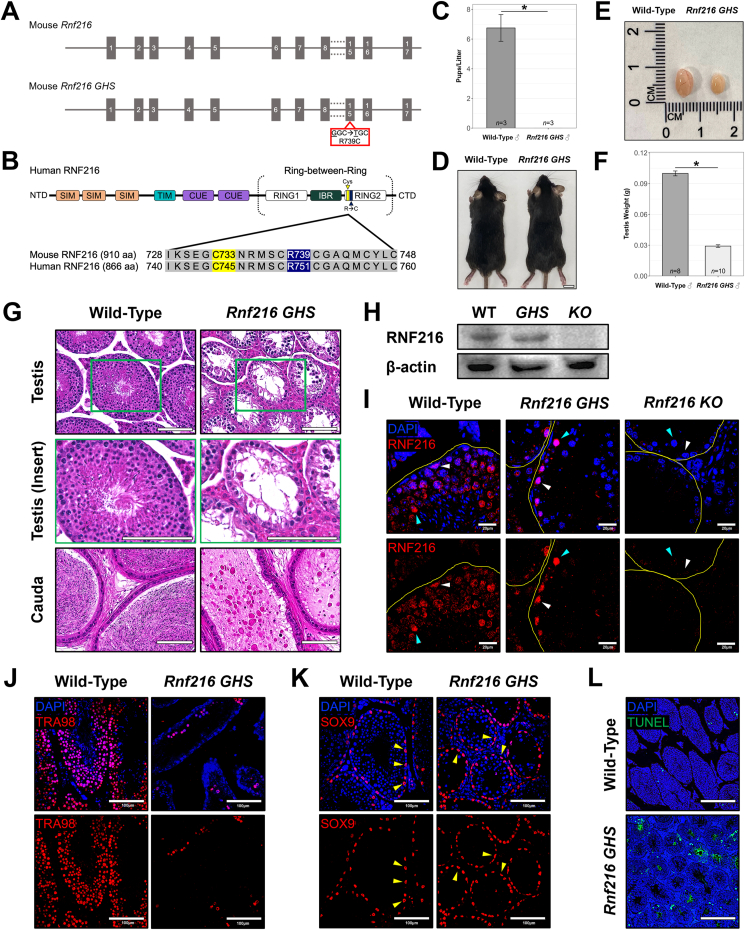
Human GHS RNF216 mutation disrupts spermatogenesis and causes infertility in male mice. **(A)** Gene map for wild-type mouse *Rnf216* and *Rnf216 GHS (Rnf216 R739C/R739C)* loci with human GHS point mutation introduced into mouse *Rnf216* exon 15 via CRISPR-Cas9. **(B)** Protein architecture for human RNF216 showing the catalytically active Ring-between-Ring (RBR) region required for E3 ubiquitin ligase activity. Catalytically active cysteine (yellow) is six residues upstream from the human GHS mutation residue (mouse p.R739C, blue). SIM, SUMO-interacting motif; TIM, TRAF-interacting motif; CUE, coupling of ubiquitin conjugation to ER degradation domain; RING, really interesting new gene; IBR, in-between-RING. Protein BLAST alignment for human and mouse RNF216, below protein diagram, showing catalytically active cysteine residue (yellow) and human GHS mutation residue (p.R→C) (blue) are conserved within the RBR region with other conserved residues (grey). **(C)***Rnf216 GHS* adult males were unable to produce offspring. *n* = number of different male mice tested. ∗*p* = 0.0001. **(D)** Wild-type and *Rnf216 GHS* adult mice show no obvious body size defects. Image is representative of ≥ 3 biological repeats. **(E)** Testis size was decreased in *Rnf216 GHS* adults. Scale bars = 1 cm. Image is representative of ≥ 3 biological repeats. **(F)** Testis weight was significantly decreased in *Rnf216 GHS* adults. *n* = number of mice. ∗*p* = 3.2e-11. **(G)** Hematoxylin-eosin-stained adult testis and cauda epididymis sections for wild-type and *Rnf216 GHS* mice. Scale bars = 100 μm. Green box for testis insert. Each image is representative of ≥ 3 biological repeats. **(H)** Western blotting analysis of RNF216 in wild-type (WT), *Rnf216 GHS* (*GHS*), and *Rnf216 KO* (*KO*) 3-month testes. β-actin was used as the loading control. The image is representative of 3 biological repeats. **(I)** Confocal images of RNF216-immunostained wild-type, *Rnf216 GHS*, and *Rnf216 KO* adult testes. Scale bars = 20 μm. Yellow lines, seminiferous tubule basement membranes; white arrowheads, spermatogonia; cyan arrowheads, spermatocytes. Each image is representative of ≥ 3 biological repeats. **(J)** Confocal images of germ cell marker TRA98-immunostained 3-month wild-type and *Rnf216 GHS* testes. Scale bars = 100 μm. Each image is representative of ≥ 3 biological repeats. **(K)** Confocal images of Sertoli cell marker SOX9-immunostained 3-month wild-type and *Rnf216 GHS* testes. Scale bars = 100 μm. Yellow arrowheads, Sertoli cells. Each image is representative of ≥ 3 biological repeats. **(L)** Confocal images of TUNEL assays for 6-week wild-type and *Rnf216 GHS* testes. Scale bars = 200 μm. Each image is representative of ≥ 3 biological repeats.

Histological analysis of adult *Rnf216 GHS* testes revealed extensive germ cell loss and reduction in spermatozoa in the caudal epididymis (Fig. 1G; Fig. S3). This phenotype closely resembled the germ cell degeneration observed in *Rnf216 KO* males, suggesting that the human GHS-associated E3 ligase-inactivating mutation impairs RNF216 function in male reproduction. Germ cell degeneration in *Rnf216 GHS* began around two weeks of age and progressed at a heterogeneous rate into late adulthood (Fig. S3). To confirm that *Rnf216 GHS* mice expressed the mutant protein *in vivo* rather than producing a null protein, we performed Western blotting analysis of RNF216 in adult testes from wild-type, *Rnf216 GHS*, and *Rnf216 KO* mice (Fig. 1H). As expected, RNF216 was detected in wild-type and *Rnf216 GHS* testes but was absent in *Rnf216 KO* testes. Furthermore, immunofluorescence staining revealed that RNF216 GHS protein was expressed in specific male germ cell populations, including remaining spermatogonia and spermatocytes, and localized to the nucleus, similar to wild-type RNF216 protein (Fig. 1I). No RNF216 protein was detected in *Rnf216 KO* germ cells. These results confirmed that the *Rnf216 GHS* mutation did not abolish RNF216 protein expression or substantially alter its subcellular localization. The seminiferous tubule degeneration observed in *Rnf216 GHS* testes was due to significant germ cell loss (*p* = 0.007) (Fig. 1J; Fig. S4A). To determine whether somatic cells were affected by the *Rnf216 GHS* mutation, Sertoli cells were quantified in adult wild-type and *Rnf216 GHS* testes. Sertoli cell numbers remained unchanged despite the RNF216 E3 ligase-inactivating mutation (*p* = 0.59) (Fig. 1K; Fig. S4B). Additionally, TUNEL assays performed on 6-week-old *Rnf216 GHS* testes revealed a significant increase in apoptosis (*p* = 3.31e-11) (Fig. 1L; Fig. S4C), which correlated with the progressive germ cell degeneration observed in these mice (Fig. S3). Altogether, these data demonstrate that the human RNF216 GHS E3 ubiquitin ligase-inactivating mutation alone is sufficient to disrupt spermatogenesis *in vivo*, resulting in male infertility in *Rnf216 GHS* mice. These findings highlight a critical role for RNF216 E3 ligase activity in the etiology of human GHS.

In summary, the introduction of the human GHS-associated RNF216 E3 ubiquitin ligase-inactivating mutation into mice resulted in disrupted spermatogenesis and male infertility. This mutation led to drastic germ cell loss, indicating that RNF216 E3 ubiquitin ligase activity is essential for male fertility. These findings not only underscore the importance of RNF216-mediated ubiquitination in mammalian spermatogenesis but also recapitulate the reproductive dysfunction observed in male GHS patients.^1^ RNF216 is expressed across multiple tissues in mice, with particularly high expression in the brain and testis.^5^ In this study, GHS-related neurological symptoms were not obvious in *Rnf216 GHS* mice but could be investigated in the future using more sensitive behavioral studies. Potential consequences of RNF216 E3 ligase inactivation in the brain, particularly its impact on testis-extrinsic regulation of reproduction via the hypothalamus–pituitary–gonad axis, warrant further investigation. In parallel, germ cell-specific studies of RNF216 are needed to more precisely define its role in male reproduction and GHS causation.

This study presents the first mouse model of human GHS, establishing a direct link between RNF216-mediated ubiquitination and disease pathogenesis. While our previous work demonstrated the importance of RNF216 in male reproduction, the introduction of a single disease-causing human amino acid mutation was sufficient to recapitulate the full reproductive phenotype observed in *Rnf216 KO* males, highlighting the essential role of RNF216 E3 ubiquitin ligase activity in GHS and providing a deeper molecular insight into the disease mechanism.^5^ These findings raise important questions about the molecular substrate(s) and associated K11/K63 ubiquitination pathways targeted by RNF216 in the testis, which could further illuminate the mechanisms underlying germ cell loss via apoptosis in male mice and reproductive dysfunction in GHS patients. The *Rnf216 GHS* mouse model provides a valuable resource for investigating and validating RNF216 ubiquitination targets *in vivo* and developing future translational therapeutics, thereby contributing to a deeper understanding of both GHS and male reproductive health.

## CRediT authorship contribution statement

**Jeffrey M. Mann:** Writing – review & editing, Writing – original draft, Visualization, Validation, Investigation, Formal analysis, Data curation. **Chao Wei:** Writing – review & editing, Validation, Investigation. **Xiaoyuan Yan:** Writing – review & editing, Validation, Investigation. **Huirong Xie:** Writing – review & editing, Methodology. **Elena Y. Demireva:** Writing – review & editing, Methodology. **Chen Chen:** Writing – review & editing, Writing – original draft, Visualization, Supervision, Resources, Project administration, Funding acquisition, Conceptualization.

## Ethics statement

All animal procedures were approved by the Institutional Animal Care and Use Committee of Michigan State University (AUF 202200230). All experiments with mice were conducted ethically following institutional guidelines according to the Guide for the Care and Use of Laboratory Animals.

## Funding

Chen Chen (C.C.) was supported by grants from the 10.13039/100000057National Institute of General Medical Sciences (No. R01GM132490 and R35GM156209 to C.C.), 10.13039/100009633Eunice Kennedy Shriver National Institute of Child Health and Human Development (No. R01HD084494 to C.C.), and 10.13039/100005825National Institute of Food and Agriculture (No. MICL02690 to C.C.). Jeffrey M. Mann was supported by research awards from the Michigan State University College of Natural Science and the Cell and Molecular Biology Graduate Program (USA). Huirong Xie and Elena Y. Demireva were supported by Michigan State University Global Impact Initiative Funds (USA).

## Conflict of interests

The authors declared no competing interests.

## References

[1] Margolin D.H., Kousi M., Chan Y.M. (2013). Ataxia, dementia, and hypogonadotropism caused by disordered ubiquitination. N Engl J Med.

[2] Cotton T.R., Cobbold S.A., Bernardini J.P., Richardson L.W., Wang X.S., Lechtenberg B.C. (2022). Structural basis of K63-ubiquitin chain formation by the Gordon-Holmes syndrome RBR E3 ubiquitin ligase RNF216. Mol Cell.

[3] Seenivasan R., Hermanns T., Blyszcz T., Lammers M., Praefcke G.J.K., Hofmann K. (2019). Mechanism and chain specificity of RNF216/TRIAD3, the ubiquitin ligase mutated in Gordon Holmes syndrome. Hum Mol Genet.

[4] Schwintzer L., Aguado Roca E., Broemer M. (2019). TRIAD3/RNF216 E3 ligase specifically synthesises K63-linked ubiquitin chains and is inactivated by mutations associated with Gordon Holmes syndrome. Cell Death Discov.

[5] Melnick A.F., Gao Y., Liu J. (2019). RNF216 is essential for spermatogenesis and male fertility. Biol Reprod.

