# Supplementary material for: Human Gordon Holmes Syndrome modeling in mice reveals essential function of RNF216 ubiquitination in spermatogenesis and male fertility
Source: Genes Dis. 2026 Jan 29;13(5):102056. doi: 10.1016/j.gendis.2026.102056 (PMC13153464; doi:10.1016/j.gendis.2026.102056)
Supplement: Multimedia component 1 [file mmc1.docx]

**Supplementary Materials**

**Materials and Methods**

*Generation and Validation of Mice*

*Rnf216 GHS* (*Rnf216 R739C/R739C*) mutant mice were generated by CRISPR-Cas9 targeting of the mouse *Rnf216* locus (ENSMUSG00000045078). Wild-type NLS-Cas9 protein, synthetic single guide RNA (gRNA), and a single strand DNA oligo (ssODN) donor template were used (Synthego, CA). Protospacer (N)20 and PAM (protospacer adjacent motif) sequences corresponding to the gRNA used were 5’- AACCGCATGTCTTGCCGCTC -TGG -3’. The sequence of the ssODN is 5’- CATAGCCATTGATAGAAACTCGACAGAGGTAGCACATCTGGGCACCACAGCaGCAAGACATGCGGTTGCAGCCTTCAGACTTGAT -3’. The *Rnf216* genomic sequence, gRNA, donor template sequences, and Sanger sequencing results alignment were annotated and visualized on Benchling online platform (benchling.com). The gRNA was incubated with Cas9 protein for 5 minutes at 37°C to pre-form ribonucleoprotein (RNP) complexes. RNPs and the donor ssODN were introduced into C57BL/6J mouse zygotes using a Gene Editor electroporator (BEX CO., LTD, Tokyo, Japan). Off-target analysis was performed with CRISPOR (crispor.gi.ucsc.edu/crispor.py) for gRNA 5’- AACCGCATGTCTTGCCGCTC -TGG -3’ and revealed no predicted off-targets in exonic regions on the same chromosome (chromosome 5). No significant off-target hits were identified in exons on other chromosomes, aside from two sites with 4 mismatches in coding regions. Similarly, only one predicted off-target site with 4 mismatches was detected in an intergenic region on chromosome 5. Importantly, all predicted hits with 4 mismatches included mismatches within the critical 12 base pairs (bp) seed region adjacent to the PAM, which are poorly tolerated and substantially reduce the likelihood of cleavage and were therefore not considered for further analysis. In addition, as part of standard protocol, all targeted animals were backcrossed to wild-type C57BL/6 for at least five generations, thereby eliminating any potential CRISPR-induced off-target mutations on non-targeted chromosomes. Embryos were implanted into pseudo-pregnant recipients according to standard procedures. Two rounds of implantation produced 5 pups, with 2 male founders (*Rnf216 R739C/+)*. Only one of these founders was used in subsequent matings in this study. The genomic editing of founder offspring was assessed using polymerase chain reaction (PCR) and Sanger sequencing of the target region. The sequences of primers used for PCR genotyping are listed in **Suppl. Table 1** and were used on tail biopsies lysed with proteinase K. PCR products underwent further restriction digestion using a mixture of restriction enzyme MspA1I (R0577S, New England Biolabs) (CA/CG:CG/TG, blunt ends), CutSmart Buffer, and ddH_2_O and were incubated at 37°C for 1 hour, followed by 65°C for 20 minutes. Reaction products were run on 2% agarose gel. Wild-type allele containing the restriction site yielded two PCR products at 267 bp and 213 bp. *Rnf216 GHS* point mutation allele without the restriction site generated a single PCR product at 480 bp. PCR products were excised from agarose gel using the E.N.Z.A Gel Extraction Kit (D2500-01, Omega) following manufacturer instructions. Extracted PCR products underwent Sanger sequencing to confirm the correct mutation location using UGENE v50.0 software (Unipro). Establishment of *Rnf216 KO* (*Rnf216 -/-*) mice was previously reported [5].

*Fertility Tests*

*Rnf216 GHS* homozygous (*Rnf216 R739C/R739C*, “*Rnf216 GHS*”) and wild-type male mice were bred with wild-type female mice of a similar age for four continuous months. Three mating cages were established for each male genotype at a 1:1 male-to-female ratio. The number of litters and pups/litter for each mating cage were recorded during this time. All mice were given identical diets, access to water, and bedding materials. Identical conditions were also carried out for female fertility tests of *Rnf216 GHS* and wild-type females bred with wild-type male mice (**Suppl. Fig. 2B**).

*Histology*

Mouse testes and epididymides were harvested, washed briefly in 1 x Phosphate-Buffered Saline (PBS), fixed overnight in either 4% paraformaldehyde (PFA) or Bouin’s fixative at 4°C on a rocker, then embedded in paraffin. For morphology analysis, Bouin’s-fixed and embedded tissues were sectioned at 5 μm and stained with hematoxylin and eosin after dewaxing and rehydration. For immunofluorescence staining and TUNEL assays, 4% PFA-fixed and embedded tissues were sectioned at 5 μm.

*Immunofluorescence Staining*

Testis were fixed in 4% PFA overnight on a rocker at 4°C and then embedded in paraffin. Testis were sectioned at 5 μm and incubated overnight at 37°C, then dewaxed and rehydrated. Antigen retrieval was performed in Tris-EDTA buffer (pH 9.0) or Citrate buffer (pH 6.0). Testis sections were blocked in 5% Normal Goat Serum (NGS) for 1 hour at room temperature. Testis sections were incubated with anti-RNF216 (1:100; A304-111A, Bethyl Laboratories; RRID:AB_2621360), anti-TRA98 (1:100; ab82527, Abcam; RRID:AB_1659152), or anti-SOX9 (1:100; 82630, Cell Signaling Technology; RRID:AB_2665492) in 5% NGS at 4°C overnight. After washing with 1 x PBS, sections were incubated with Alexa Fluor 555 goat anti-rabbit IgG (1:500; A21429, Life Technologies; RRID:AB_2535850) or Alexa Fluor 555 goat anti-rat IgG (1:500; A21434, Life Technologies ; RRID:AB_141733) for 1 hour and mounted with Vectashield mounting media with DAPI. Fluorescence microscopy was performed using Fluoview FV1000 confocal microscope (Olympus).

*Western Blot*

Testes were collected and homogenized using RIPA buffer (J63306-AP, Thermo Fisher Scientific) with protease inhibitor (A32965, Thermo Fisher Scientific). Protein lysates were separated by 4–20% polyacrylamide gels (4561096, Bio-Rad) and transferred to PVDF membranes. Membranes were blocked in 5% non-fat milk at room temperature for 30 minutes and subsequently incubated with anti-RNF216 (1:1000; A304-111A, Bethyl Laboratories; RRID:AB_2621360) or anti-β-Actin−Peroxidase (1:5000, A3854, Sigma; RRID:AB_262011) in 5% non-fat milk at 4°C overnight. Membranes were washed with 1 x Tris-Buffered Saline with 0.1% Tween 20 (TBS-T) and incubated with HRP-conjugated goat anti-rabbit IgG (1:5000; 1706515, Bio-Rad; RRID:AB_11125142) at room temperature for 1 hour followed by chemiluminescent detection with ECL Substrate (1705060, Bio-Rad).

*TUNEL Assay*

*In Situ* Cell Death Detection Kit - Fluorescein (11684795910, Roche) was used to evaluate cell death in 4% PFA-fixed paraffin-embedded 3-month testes sections according to manufacturer’s instructions. Fluorescence images were captured by Fluoview FV1000 confocal microscope (Olympus).

*Statistical Analysis*

All statistical analyses were conducted unblinded using R Statistical Software (version 4.5.0). Data were visualized using the ggplot2 package. All data are presented as mean±SEM. The one sample or two sample *t* test was used for statistical analyses, when appropriate. Statistical significance (*) was determined by *p*<0.01.


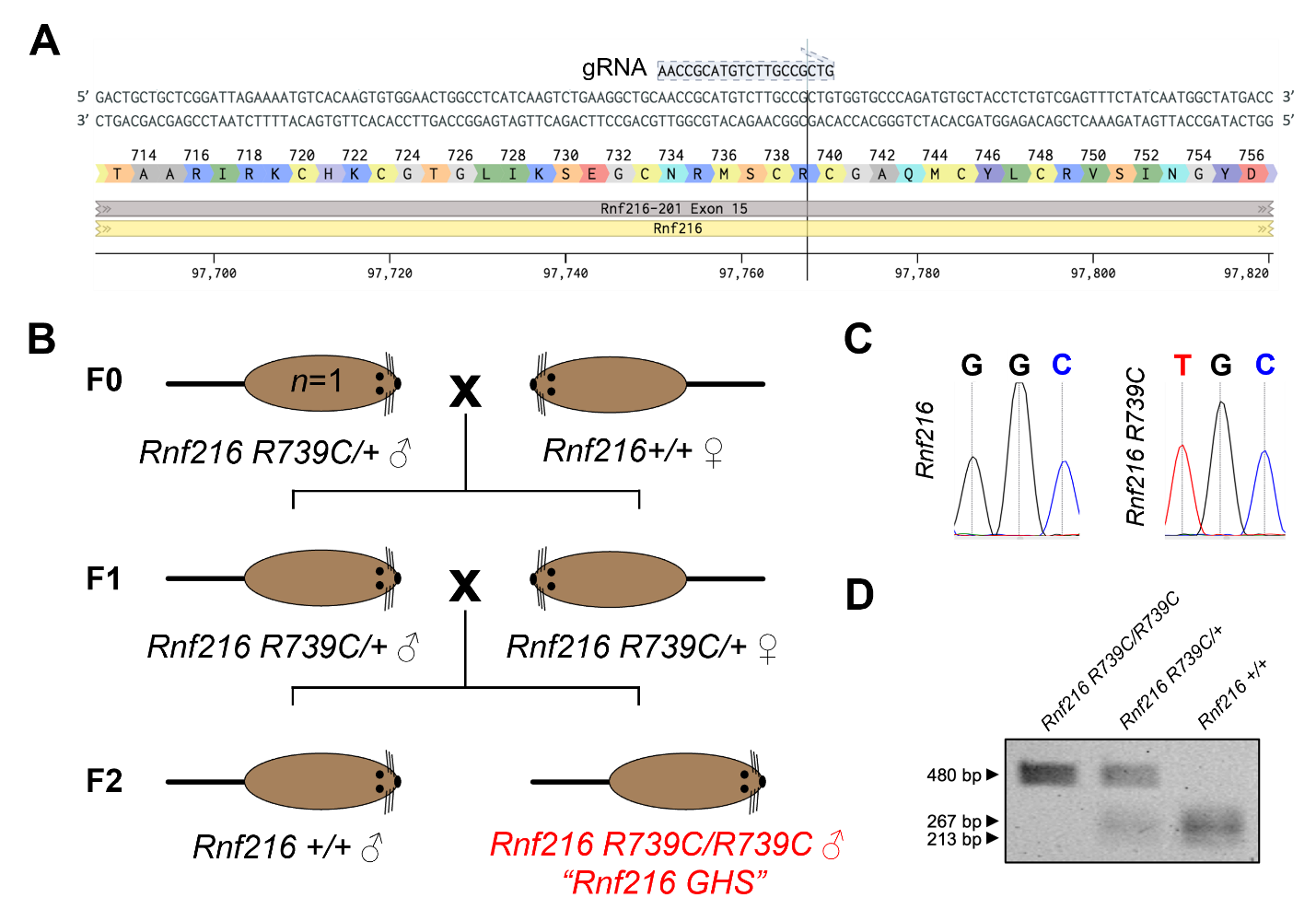


**Supplemental Figure 1. Generation and validation of *Rnf216 GHS* mice.**

**A.** Strategy for generation of *Rnf216 GHS* (*Rnf216 R739C/R739C*) mice using gRNA, ssODN donor template, and CRISPR-Cas9 to introduce human GHS point mutation into mouse *Rnf216* exon 15 (p.R739🡪C). **B.** Mating schematic to generate homozygous F2 *Rnf216 R739C/R739C* (red) (*Rnf216 GHS*) mice using F1 heterozygotes derived from a male F0 founder (*n*=1), *n*=number of founder mice used. **C.** Wild-type (*Rnf216*) and *Rnf216 GHS* allele Sanger sequencing results, respectively, confirming correct human GHS point mutation in mice. **D.** Representation of PCR and restriction digestion used to genotype *Rnf216 GHS* mice using agarose gel electrophoresis.

**
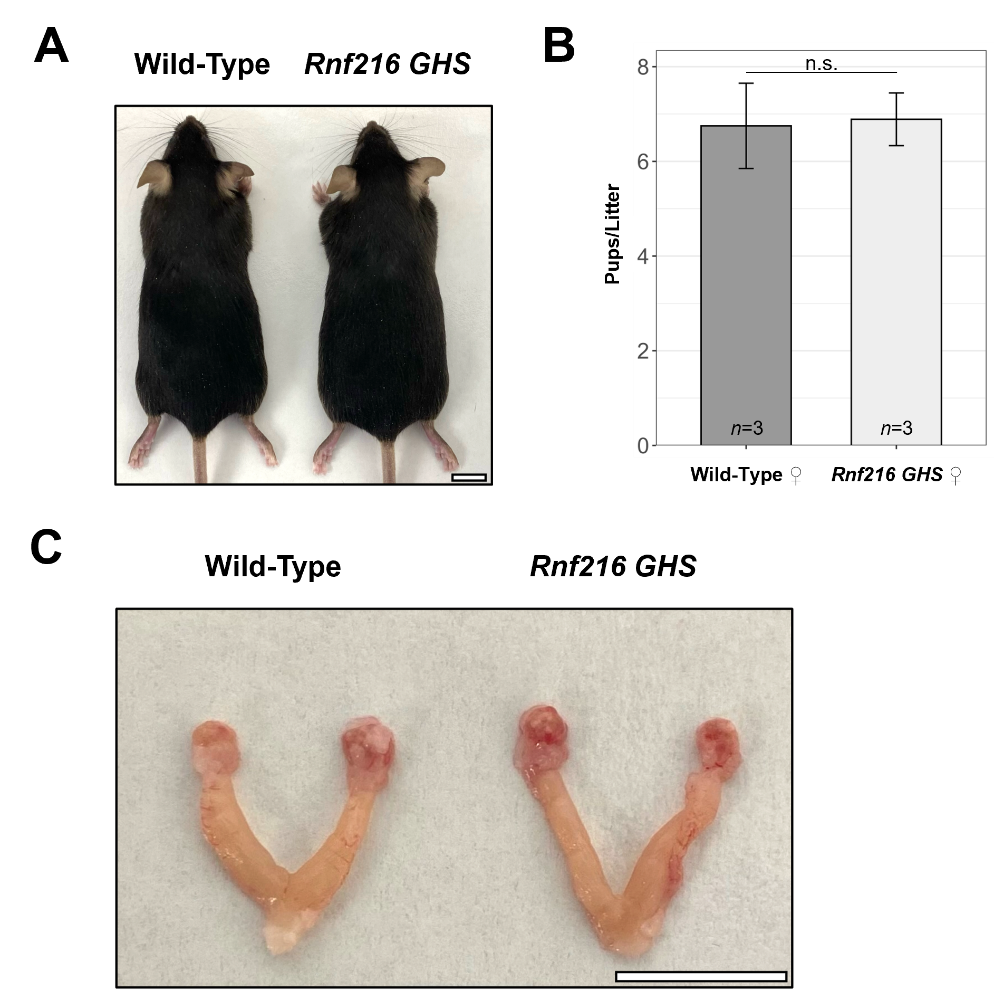
**

**Supplemental Figure 2. Examination of *Rnf216 GHS* female mice.**

**A.** No obvious body size difference between female wild-type (*n*=3) and *Rnf216 GHS* adult (*n*=3) (3-month) mice, *n*=number of mice, scale bar 1 cm. **B.** *Rnf216 GHS* adult females were fertile and produced offspring, *n*=number of female mice tested, n.s.=non-significant. **C.** No obvious difference between female wild-type (*n*=3) and *Rnf216 GHS* (*n*=3) adult uteri and ovaries, *n*=number of mice, scale bar 1 cm.

**
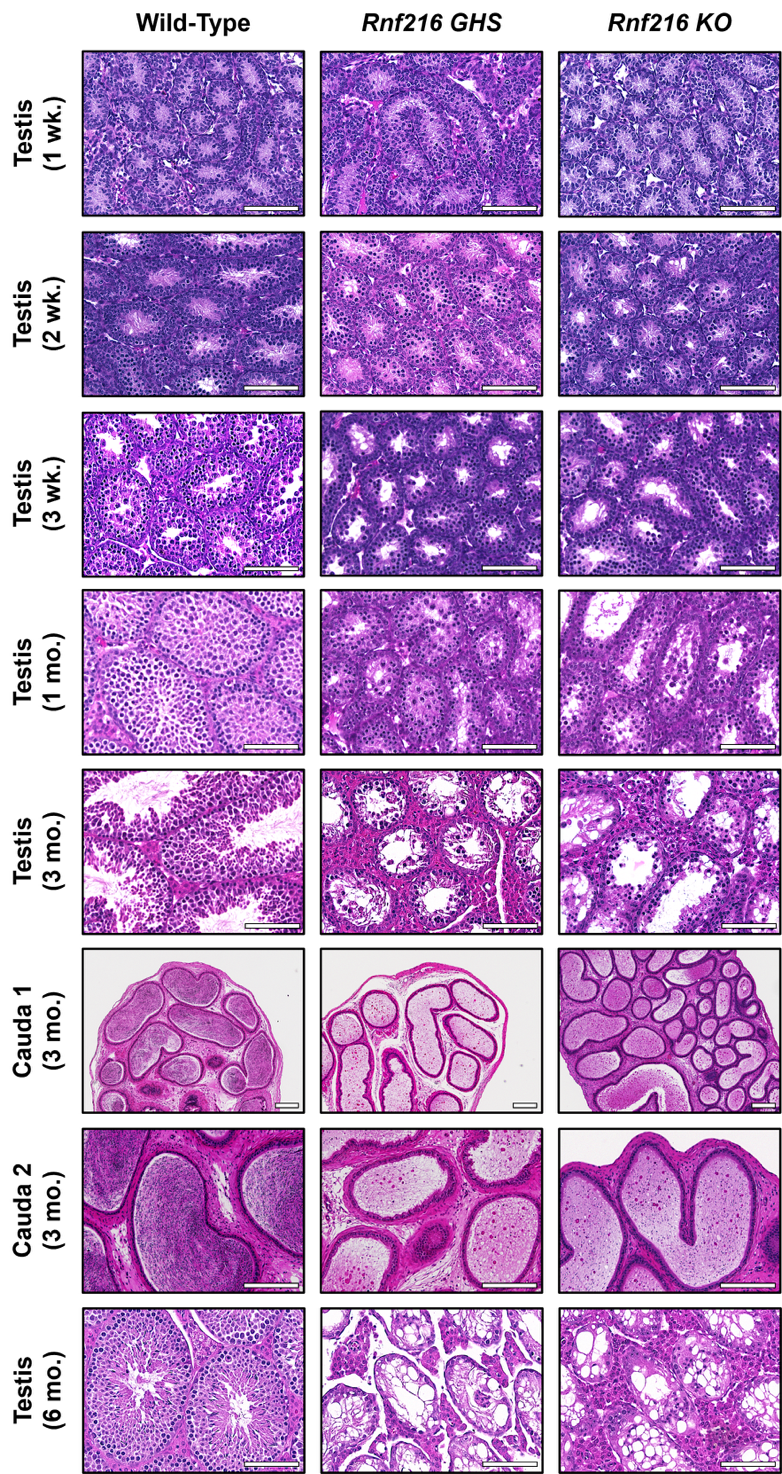
**

**Supplemental Figure 3. Expanded histology panel of testes and epididymides at various ages*.***

Light microscope images of hematoxylin and eosin-stained testes and caudal epididymides at increasing ages of wild-type, *Rnf216 GHS*, and *Rnf216 KO* male mice. Each image is representative of ≥3 biological repeats, scale bars 200 µm.


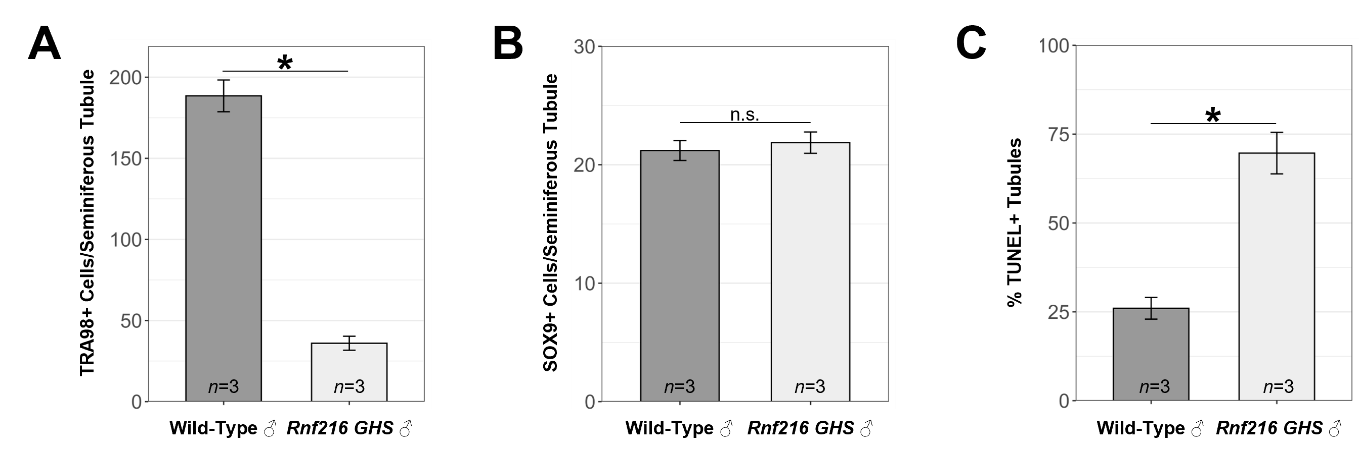


**Supplemental Figure 4. Quantification of germ cells, Sertoli cells, and TUNEL-positive seminiferous tubules.**

**A.** TRA98-positive germ cells were quantified per seminiferous tubule cross-section in wild-type and *Rnf216 GHS* 3-month mouse testes (*n*=3), *n*=number of mice, **p*<0.01. **B.** SOX9-positive Sertoli cells were quantified per seminiferous tubule cross-section in wild-type and *Rnf216 GHS* 3-month mouse testes (*n*=3), *n*=number of mice, n.s.=non-significant. **C.** Percentage of TUNEL-positive seminiferous tubule cross-sections was quantified in wild-type and *Rnf216 GHS* 3-month mice testes (*n*=3), *n*=number of mice, **p*<0.01.

| **Gene** | **Direction** | **Primer Sequence** |
| --- | --- | --- |
| *Rnf216 GHS* | Forward | 5’-ATGTCTCGGAAAGGCTCAGG-3’ |
|  | Reverse | 5’-CTCCGTTTTCCCTGTGGCTA-3’ |
| *Rnf216 KO* | Forward | 5’-GGCGCATAACGATACCACGA-3’ |
|  | Reverse | 5’-AAGCCAAATCAGAGGACGGG-3’ |

**Supplementary Table 1. PCR primer sequences for *Rnf216* mouse line genotyping.**
